# Evaluation of the Olympus Demand random access chemistry analyser

**DOI:** 10.1155/S1463924687000257

**Published:** 1987

**Authors:** Susan B. Schotters, James H. McBride, Denis O. Rodgerson, Margaret H. McGinley, Marilyn Pisa

**Affiliations:** Clinical Chemistry Laboratory Department of Pathology UCLA School of Medicine Los Angeles California 90024 USA


Journal of Automatic Chemistry/*Journal of Clinical Laboratory Automation*, Vol. 9, No. 3 (July–September 1987), pp. 113–118

### Evaluation of the Olympus Demand random access chemistry analyser

**Susan B. Schotters, James H. McBride, Denis O. Rodgerson, Margaret H. McGinley and Marilyn Pisa**

*Clinical Chemistry Laboratory, Department of Pathology, UCLA School of Medicine, Los Angeles, California 90024, USA*

*We evaluated the Olympus Demand as a cost-saving measure, as a back up to a Technicon SMAC I, and to be the primary instrument for enzyme analysis. In general, it was found to be precise, reliable, easy to operate, but with only average turn-around time capabilities. Technically there were several limitations: (1) significant bilirubin interference in the analysis of serum bicarbonate and creatinine; (2) instability of the ion selective electrodes; (3) lengthy routine maintenance required for the ion selective electrode module; (4) serum phosphorus and uric acid methods required blanking due to interference from bilirubin, hemolysis, or lipemia; and (5) serum triglyceride analysis included measurement of free glycerol.*

The Olympus Demand model AU 500 (Cooper Biomedical Inc., Malvern, Pennsylvania 19355, USA) was selected for evaluation from among several chemistry discrete analysers available on the market. The instrument which is a fully automated random access analyser, is capable of handling up to 100 samples for 23 chemistry tests at any given time. It comprises an analytical and a control console, both of which are connected by a communication cable, permitting some flexibility in their arrangement. The maximum throughput is 400 tests per hour, which can be increased if sodium, potassium, and chloride are included.

A primary goal of the evaluation was to achieve improved cost effectiveness by replacing the use of one of two Technicon SMAC Is (Technicon Instruments Corporation, Tarrytown, New York 10591, USA) and provide back up for the remaining SMAC I. Improved cost effectiveness could be obtained by decreasing technologist time, cost of reagents (discrete testing versus continuous flow), and numerous repeat analysis due to channel malfunction. Two limited evaluations of the Demand have since confirmed cost effectiveness of the instrument [1 and 2]. Another major goal of the evaluation was to investigate an alternative instrument for measurement of enzyme activities.

### Materials and methods

### *Instrument operation*

The Demand uses equilibrium, kinetic, and ion selective electrodes for measurement methods. Equilibrium methods are based on single point calibration which is stable for 24 h with the exception of bicarbonate which must be recalibrated every 4 h. Kinetic measurements are based on preprogrammed molar absorptivity values. If substrate depletion occurs, it is detected and indicated by the instrument. Ion selective electrodes (ISE) use two-point internal calibration with an optional external calibrator. The ISE calibrates automatically every 30 min and in addition calibrates whenever a run of samples includes a sodium, potassium or chloride measurement.

The analytical console contains a refrigerated reagent turntable, fluids metering system, sampler, cuvette loader, cuvette turntable, bichromatic photometric measuring system (10 wavelengths available), and the ISE module. All temperatures are automatically controlled and an alarm system is present to detect any deviation. The control console, which commands the analytical unit, consists of a data processor unit (DPU), cathode ray tube (CRT), keyboard, and printer. The Demand has bidirectional computer capabilities but requires an interface board in the DPU, appropriate software for the host computer, and an Apple IIe personal computer (Apple Computer, Cupertino, California 95014, USA).

The control console is used to program tests to be performed on each sample and the capacity for programming is 100 samples excluding calibrators and controls. Once the analyses is completed on a sample, that sample 'position' is again available for programming of tests requested for a new sample.

The Demand uses a variety of colour-coded sample cup carriers to contain patient specimens, controls, or calibrators. As the sample carrier chain is advanced to the sample probe area, the carrier type and position are read. This information is matched with the previously programmed tests requested and directs the loading of the correct number of cuvettes, sample volumes and reagent volumes. The plastic disposable cuvettes are loaded onto the 72-position turntable every 9 s as it rotates and all samples and reagents are diluted with prewarmed deionized water and mechanically mixed. Before the sample is added, a set of absorbance readings is taken which serve as a reagent blank. After the sample is added, a set of absorbances is read at the primary and secondary wavelengths followed by a second set of absorbances taken at a later photometer. This information is transmitted to the DPU; and when all analyses are completed, the results are printed. Also, the instrument has the capability of printing absorbance units.

Daily and weekly maintenance is minimal, partly due to the fact that the sample and two reagent probes are washed internally and externally after each use. The ISE module requires more time for maintenance, especially the weekly procedure.

*Table 1. Manufacturer's recommended methods, reagent volumes, and sample volumes for the Olympus Demand.*

| Test | Method | Primary/second wavelength | Sample volume (μl) | Reagent volume (μl) |
|---|---|---|---|---|
| Sodium | | | | |
| Potassium | Ion selective electrodes | Not applicable | 50 | 20 |
| Bicarbonate | Phosphoenolpyruvate carboxylase – equilibrium | 380/410 | 7 | 70 |
| Glucose | Hexokinase – UV equilibrium | 340/380 | 5 | 100 |
| Creatinine | Jaffe – kinetic | 520/570 | 25 | 40 |
| BUN | Urease – kinetic | 340/380 | 10 | 60 |
| Total protein | Biuret – equilibrium | 540/660 | 10 | 100 |
| Albumin | Bromcresol green – equilibrium | 600/540 | 5 | 100 |
| Inorganic phosphorus | Ammonium molybdate – UV equilibrium | 340/380 | 10 | 100 |
| Total bilirubin | Diazotized sulphanilic acid – equilibrium | 540/600 | 15 – Blank<br>15 – Test | 60 – Blank<br>60 – Test |
| Conjugated bilirubin | Diazotized sulphanilic acid – equilibrium | 540/600 | 15 – Blank<br>15 – Test | 60 – Blank<br>60 – Test |
| ALP | p-Nitrophenylphosphate – kinetic | 410/480 | 7 | 100 |
| AST | Oxaloacetate/NADH – UV kinetic | 340/380 | 15 | 50 |
| ALT | Pyruvate/NADH – UV kinetic | 340/380 | 25 | 30 |
| LD | Lactate/$NAD^+$ – UV kinetic | 340/380 | 10 | 50 |
| CK | Glucose-6-phosphate/$NADP^+$–UV kinetic | 340/380 | 15 | 50 |
| Cholesterol | Cholesterol esterase/oxidate – equilibrium | 540/600 | 5 | 100 |
| Triglyceride | Tetrazolium chloride/NADH – equilibrium | 520/600 | 5 | 50 |
| Uric acid | Uricase/quinonemine dye –equilibrium | 540/600 | 20 | 60 |

### Reagents and calibrators

The tests evaluated in our laboratory using Cooper Biomedical reagents were sodium, potassium, chloride, bicarbonate, glucose, creatinine, BUN (blood urea nitrogen), total protein, albumin, inorganic phosphorus, total bilirubin, conjugated (direct) bilirubin, ALP, ASP, ALT, LD, CK, cholesterol, trigylceride, and uric acid*. All analyses were carried out at 37 °C in accordance with manufacturer's instructions [3]. Table 1 provides a summary of methods, sample volumes, and reagent volumes. The instrument can be operated with user-defined methods [4].

Initially, the Cooper Biomedical protein based calibrator, Flozyme, was used for all tests except conjugated bilirubin and the ISE. Beckman Direct Bilirubin Calibrator Number 7 (Beckman Instruments, Inc., Brea, California 92621, USA) was used for the conjugated bilirubin and the ISE were calibrated with two levels of internal calibrators. Due to the low concentration of total bilirubin in Flozyme, a change was made to Omega Chemistry Control Serum-Elevated Bilirubin (Cooper Biomedical, Inc, Malvern, Pennsylvania 19355, USA) for the total bilirubin calibrator. All calibration material concentrations were verified with primary standards where possible.

To assess photometric precision, a solution of potassium dichromate (4·2 g/l) was prepared and analysed on the instrument at wavelengths 380/410 nm comparing the first photometer with the other 14 photometers. With-in run and between-run precision studies were performed using a combination of three different control materials at various concentrations. The control materials were Omega Assayed Control Serum I and II, Omega Chemistry Control Serum-Elevated Bilirubin, and Technicon TQC Chemistry Control L and H (Technicon Instruments Corporation, Tarrytown, New York 10951, USA).

To study carry-over of the sample dispenser, two glucose solutions containing 4·0 mmol/l and 31·6 mmol/l were prepared. These solutions were analysed alternatively ($N$ = 20) and percentage carry-over determined [5].

Linearity was evaluated using primary aqueous standards prepared in our laboratory for sodium, chloride, bicarbonate, glucose, BUN, inorganic phosphorus, and uric acid. For potassium and creatinine the Linearity Multi-Point Standard Set (American Scientific Products, McGaw Park, Illinois 60085, USA) was used. Two specially prepared albumin-based materials from Clinical Chemistry Consultants (Temple City, California 91780, USA) were obtained to test the linearity of ALP, AST, ALT, LD, CK, and cholesterol. Protein Standard Solution (Sigma Chemical Company, St. Louis, Minnesota 63178, USA) was utilized for the total protein and

* Non-standard abbreviations: ALP, alkaline phosphatase (EC 3.1.3.1); AST, aspartate aminotransferase (EC 2.6.1.1); ALT, alanine aminotransferase (EC 2.6.1.2); CK, creatine kinase (EC 2.7.3.2); LD, lactate dehydrogenase (EC 1.1.1.27).

Normal Serum Albumin (Human) 25% (Cutter Biological, Berkeley, California 94710, USA) was used for albumin linearity. Crystalline bilirubin (Sigma Chemical Co., St. Louis, Minnesota 63178, USA) was used for total bilirubin and pooled patient serum was employed for conjugated bilirubin linearity evaluation. For the linearity testing of triglyceride, a patient body fluid with a concentration of greater than 28·2 mmol/l was utilized. Each linearity study was performed with no less than five levels of concentration; each level analysed in triplicate, and a mean value determined.

For the correlation study we used patient sera that had been analysed on the same day on SMAC I. All specimens were kept covered to minimize evaporation between assays.

The effect of bilirubin, hemolysis, and lipemia on all analytes was investigated. For bilirubin, selected serum samples containing a wide concentration range of each analyte were spiked with crystalline bilirubin at a concentration of 171 µmol/l dissolved in a normal pooled serum. Aliquots of the same selected samples were spiked with three levels of concentration of hemoglobin 500·0 g/l, 2500·0 g/l, and 5000·0 g/l for the hemolysis study. In both of these an aliquot of the nonspiked serum sample was diluted appropriately to compensate for the dilutional effects of the spiking. For the lipemia study serum samples were selected which visually had slight (1+) to gross lipemia (4+) present. These samples were analysed before and after ultracentrifugation in a Beckman Airfuge (Beckman Instruments, Inc., Brea, California 92621).

### Results

### *Precision*

Within-run precision ($N$ = 14) of the 14 photometers resulted in a CV of ±1·0%. Within-run precision ($N$ = 20) was performed on all tests using three different control materials at various concentrations. The between-run precision studies were performed using the same control materials over a period of 20 days. Table 2 shows both the within-run and between-run precision results of all analytes. BUN was the only analyte to have a within-run precision CV greater than ±5%, however, that was at a concentration level of 1·4 mmol/l. At the same concentration for BUN, the between-run precision had the highest CV at ±11·3%. Bicarbonate was found to have a between-run CV of ±8·0/ at a level of 10 mmol/l.

### *Carry-over*

Sample to sample carry-over was 0·49%.

### *Linearity*

All but two of the analytes, BUN and total protein, met the manufacturer's stated range of linearity (table 3). BUN was linear to 32·1 mmol/l versus the stated value of 35·7 mmol/l and the total protein was linear to 80·0 g/l (stated linearity 140·0 g/l), but the study was limited by the lack of material at a higher protein concentration.

### *Correlation*

For each analyte, 74 to 128 serum samples which had been analysed on SMAC I were run on the Demand with the exception of cholesterol where 36 serum samples were correlated. The original cholesterol correlation had included 90 samples but the study had to be repeated upon notification from the manufacturer that the original assigned calibrator value was changed from 4·0 mmol/l to 4·4 mmol/l. The range of slope values was 0·9228 to 1·0844 with the exception of lower slope values for ALP, ALT, and LD. Table 4 lists the range of concentrations the actual number of samples tested for each analyte and the linear regression equations obtained.

### *Interference studies*

Interference caused by bilirubin concentrations greater than 171·0 µmol/l significantly increased bicarbonate values and decreased creatinine, phosphorus and cholesterol values. Samples with slight hemolysis (approximately 500·0 g/l) had significantly increased potassium and LD, and decreased phosphorus; whereas moderate hemolysis (approximately 2500·0 g/l) significantly increased potassium, AST, and CK. Excessive increases for bicarbonate, LD, and total protein and decreases for phosphorus and albumin caused by moderate hemolysis rendered those samples unsuitable for analysis.

Slight lipemia resulted in significant increases in glucose, total protein, and phosphorus. Moderate to gross lipemia increased glucose, total protein, phosphorus, total bilirubin and conjugated bilirubin. Any trace of lipemia increased uric acid concentrations so greatly that the analysis was deemed unacceptable until the method was modified by sample blanking and adjustment of the secondary wavelength from 600 nm to 660 nm. Moderate to gross lipemia significantly decreased the concentration of sodium, potassium, chloride, bicarbonate, creatinine, BUN, albumin, ALP, AST, ALT, LD, and CK. Ultracentrifugation eliminated the lipemia interference but would not be appropriate for samples to be tested for total bilirubin and conjugated bilirubin, cholesterol, and triglyceride. Interferences from hemolysis, bilirubin, and lipemia with the Demand have recently been reported by Glick *et al.* [6].

### Discussion

Essentially, the Demand has several valuable technical features which include flexibility in the choice of test selection, methods, and source of reagents. Other features include sample and reagent metering systems regulated by a digitally controlled stepping motor. Also, there is negligible sample-to-sample or reagent-to-reagent carry-over. Further, the Demand uses small reagent vials requiring minimal refrigerated storage space, and a refrigerated turntable is included in the analyser console. Other useful characteristics include liquid-level detectors for both samples and reagents, built-in temperature control and alarms, little day-to-day maintenance, and no requirement for external plumbing. A diagnostic program is included in the software which helps in trouble-shooting and correction of malfunctions.

*Table 2. Precision of the Olympus Demand.*

| | | | | Within-run | | | All levels $N$ = 20 | | | |
|---|---|---|---|---|---|---|---|---|---|---|
| | | Level 1 | | | Level 2 | | | Level 3 | | |
| Test | | Mean | SD | CV% | Mean | SD | CV% | Mean | SD | CV% |
| Sodium | mmol/l | 98·8 | 0·77 | 0·78 | 124·1 | 0·63 | 0·51 | 147·6 | 1·24 | 0·84 |
| Potassium | mmol/l | 2·2 | 0·04 | 1·88 | 5·0 | 0·05 | 0·88 | 8·0 | 0·09 | 1·04 |
| Chloride | mmol/l | 79·9 | 0·94 | 1·17 | 97·1 | 0·81 | 0·83 | 120·1 | 1·1 | 0·92 |
| Bicarbonate | mmol/l | 9·9 | 0·31 | 3·10 | 20·3 | 0·49 | 2·38 | 30·6 | 0·6 | 1·96 |
| Glucose | mmol/l | 2·4 | 0·03 | 1·29 | 5·8 | 0·14 | 2·34 | 16·7 | 0·23 | 1·38 |
| Creatinine | µmol/l | 80·4 | 2·21 | 2·48 | 429·0 | 7·10 | 1·52 | — | — | — |
| BUN | mmol/l | 1·5 | 0·08 | 5·52 | 5·1 | 0·18 | 3·46 | 18·2 | 0·32 | 1·75 |
| Total Protein | g/l | 45·6 | 1·20 | 2·51 | 79·1 | 1·00 | 1·29 | — | — | — |
| Albumin | g/l | 20·3 | 0·50 | 2·32 | 41·7 | 0·70 | 1·55 | — | — | — |
| Inorganic Phosphorus | mmol/l | 1·4 | 0·03 | 2·18 | 2·4 | 0·03 | 1·33 | — | — | — |
| Total Bilirubin | µmol/l | 15·6 | 0·51 | 3·39 | 86·7 | 1·20 | 1·30 | 258·9 | 2·22 | 0·87 |
| Conjugated Bilirubin | µmol/l | 170·8 | 1·54 | 0·92 | — | — | — | — | — | — |
| ALP | U/l | 90·8 | 2·20 | 2·32 | 242·1 | 4·80 | 1·99 | — | — | — |
| AST | U/l | 27·0 | 0·86 | 3·18 | 93·1 | 1·40 | 1·50 | — | — | — |
| ALT | U/l | 22·2 | 0·84 | 3·78 | 79·7 | 0·82 | 1·02 | — | — | — |
| LD | U/l | 143·4 | 2·91 | 2·03 | 369·7 | 4·93 | 1·24 | — | — | — |
| CK | U/l | 105·4 | 1·57 | 1·49 | 398·7 | 2·48 | 0·62 | — | — | — |
| Cholesterol | mmol/l | 4·1 | 0·10 | 2·46 | 6·8 | 0·12 | 1·73 | — | — | — |
| Triglyceride | mmol/l | 1·1 | 0·04 | 3·43 | 3·5 | 0·07 | 1·93 | — | — | — |
| Uric acid | µmol/l | 279·0 | 7·14 | 2·49 | 469·9 | 5·95 | 1·23 | — | — | — |

| | | | | Between-run | | | All levels $N$ = 20 | | | |
|---|---|---|---|---|---|---|---|---|---|---|
| | | Level 1 | | | Level 2 | | | Level 3 | | |
| Test | | Mean | SD | CV% | Mean | SD | CV% | Mean | SD | CV% |
| Sodium | mmol/l | 99·7 | 1·19 | 1·20 | 123·9 | 2·21 | 1·79 | 147·6 | 1·64 | 1·11 |
| Potassium | mmol/l | 2·3 | 0·08 | 3·51 | 5·1 | 0·11 | 1·79 | 8·1 | 0·16 | 2·00 |
| Chloride | mmol/l | 76·1 | 0·97 | 1·27 | 98·8 | 2·07 | 2.10 | 120·7 | 1·70 | 1·41 |
| Bicarbonate | mmol/l | 10·2 | 0·82 | 8·01 | 19·0 | 1·27 | 6·36 | 30·5 | 1·75 | 5·72 |
| Glucose | mmol/l | 2·4 | 0·05 | 2·08 | 5·6 | 0·16 | 2·92 | 16·2 | 0·09 | 4·54 |
| Creatinine | µmol/l | 79·6 | 0·00 | 0·00 | 414·6 | 88·40 | 2·12 | — | — | — |
| BUN | mmol/l | 1·6 | 0·19 | 11·34 | 5·0 | 0·25 | 5·06 | 18·0 | 0·54 | 3·03 |
| Total Protein | g/l | 44·4 | 1·30 | 2·83 | 80·3 | 2·30 | 2·83 | — | — | — |
| Albumin | g/l | 21·2 | 0·60 | 2·78 | 39·6 | 1·10 | 2·62 | — | — | — |
| Inorganic Phosphorus | g/l | 1·3 | 0·05 | 3·41 | 2·5 | 0·07 | 2·74 | — | — | — |
| Total Bilirubin | µmol/l | 14·9 | 0·86 | 5·33 | 84·8 | 1·20 | 1·33 | 258·9 | 3·08 | 2·20 |
| Conjugated Bilirubin | µmol/l | 169·8 | 3·25 | 1·93 | — | — | — | — | — | — |
| ALP | U/l | 80·6 | 3·54 | 4·39 | 223·5 | 6·22 | 2·79 | — | — | — |
| AST | U/l | 25·4 | 1·26 | 4·95 | 89·6 | 2·02 | 2·25 | — | — | — |
| ALT | U/l | 22·3 | 1·09 | 4·86 | 80·1 | 2·00 | 2·50 | — | — | — |
| LD | U/l | 135·6 | 6·27 | 4·73 | 374·0 | 9·10 | 2·43 | — | — | — |
| CK | U/l | 100·3 | 6·84 | 6·82 | 399·7 | 16·86 | 4·22 | — | — | — |
| Cholesterol | mmol/l | 3·8 | 0·12 | 3·24 | 6·5 | 0·16 | 2·42 | — | — | — |
| Triglyceride | mmol/l | 1·1 | 0·04 | 3·36 | 3·3 | 0·06 | 1·86 | — | — | — |
| Uric acid | µmol/l | 276·6 | 10·71 | 3·76 | 472·3 | 17·25 | 3·64 | — | — | — |

After evaluation of the Demand, it was put into routine operation replacing one SMAC I, providing back up for the remaining SMAC I, and becoming the primary instrument for enzyme analysis. The main cost-saving benefit came from the major decrease in reagent use and reduction in repeat analysis due to channel malfunctions of SMAC I. However, the decrease in technologist time was not fully realized due to the slow turn-around time for urgent measurement of electrolytes and the multiple technical problems. It has proven, on the whole, to be a reliable instrument and has held up well with 24 hours a day, seven days a week useage. Over the last two years the down time has been minimal (average of 40 hours per year). This last fact is, in part, attributable to the diagnostic program included in the instrument's software.

*Table 3. Linearity of the Olympus Demand.*

| Test | Material | Determined range of linearity | | Stated range of linearity | |
|---|---|---|---|---|---|
| Sodium | Primary aqueous standard | 70–200 | mmol/l | 80–199 | mmol/l |
| Potassium | Multi-point linearity standards/scientific products | 1·5–10·0 | mmol/l | 2·5–9·9 | mmol/l |
| Chloride | Primary aqueous standard | 70–199 | mmol/l | 70–199 | mmol/l |
| Bicarbonate | Primary aqueous standard | 0–50 | mmol/l | 0–50 | mmol/l |
| Glucose | Primary aqueous standard | 0–41·6 | mmol/l | 0–41·6 | mmol/l |
| Creatinine | Multi-point linearity standards/scientific products | 0–2210 | μmol/l | 0–2210 | μmol/l |
| BUN | Primary aqueous standard | 0–32·1 | mmol/l | 0–35·7 | mmol/l |
| Total protein | Protein standard solution/Sigma Chemical Company | 0–80·0 | g/l | 0–140·0 | g/l |
| Albumin | Serum albumin (human) 25%/Cutter Biological | 0–60·0 | g/l | 0–80·0 | g/l |
| Inorganic phosphorus | Primary aqueous standard | 0–5·2 | mmol/l | 0–5·2 | mmol/l |
| Total bilirubin | Bilirubin/Sigma Chemical Company | 0·513·0 | μmol/l | 0–513·0 | μmol/l |
| Conjugated bilirubin | Pooled patient serum specially prepared albumin based | 0–205·2 | μmol/l | 0–205·2 | μmol/l |
| ALP | Material/Clin. Chem. Consultants | 0·1500 | U/l | 0–1500 | U/l |
| AST | " | 0–600 | U/l | 0–600 | U/l |
| ALT | " | 0–500 | U/l | 0–500 | U/l |
| LD | " | 0–2400 | U/l | 0–2400 | U/l |
| CK | " | 0–1500 | U/l | 0–1500 | U/l |
| Cholesterol | " | 0–12·9 | mmol/l | 0–12·9 | mmol/l |
| Triglyceride | Patient body fluid with >28·2 mmol/l of triglyceride concentration | 0–6·8 | mmol/l | 0–6·8 | mmol/l |
| Uric acid | Primary aqueous standard | 0–1189·6 | μmol/l | 0–1189·6 | μmol/l |

*Table 4. Correlation of the Olympus Demand with SMAC I.*

| Test | Range of concentration | | *N* | SMAC I mean | Demand mean | Slope | Y-intercept | Correlation coefficient |
|---|---|---|---|---|---|---|---|---|
| Sodium | 97–178 | mmol/l | 74 | 136·7 | 136·2 | 0·9858 | 1·43 | 0·9876 |
| Potassium | 1·2–7·0 | mmol/l | 124 | 4·4 | 4·4 | 0·9478 | 0·19 | 0·9946 |
| Chloride | 85–131 | mmol/l | 94 | 104·8 | 105·6 | 0·9915 | 1·74 | 0·9528 |
| Bicarbonate | 13–40 | mmol/l | 85 | 25·1 | 24·5 | 0·9228 | 1·31 | 0·9571 |
| Glucose | 3·8–33·1 | mmol/l | 97 | 8·4 | 8·5 | 1·0114 | − 0·04 | 0·9991 |
| Creatinine | 44·2–1785·7 | μmol/l | 128 | 326·2 | 312·1 | 0·9415 | 4·60 | 0·9984 |
| BUN | 1·8–41·4 | mmol/l | 120 | 13·2 | 13·0 | 0·9791 | 0·27 | 0·9973 |
| Total Protein | 48·0–97·0 | g/l | 75 | 69·0 | 70·3 | 1·0229 | − 0·30 | 0·9857 |
| Albumin | 15·0–56·0 | g/l | 78 | 49·4 | 44·4 | 0·9378 | 6·00 | 0·9705 |
| Inorganic phosphorus | 5·5–60·4 | mmol/l | 80 | 1·3 | 1·4 | 1·0082 | 0·06 | 0·9850 |
| Total bilirubin | 3·4–451·4 | μmol/l | 93 | 61·6 | 63·3 | 1·0770 | − 2·57 | 0·9956 |
| Conjugated bilirubin | 1·7–256·5 | μmol/l | 73 | 20·3 | 19·4 | 0·9045 | 1·20 | 0·9804 |
| ALP | 31–2180 | U/l | 108 | 244·2 | 168·4 | 0·5935 | 23·51 | 0·9901 |
| AST | 8–1430 | U/l | 91 | 100·8 | 78·9 | 0·9604 | − 17·95 | 0·9829 |
| ALT | 6–1550 | U/l | 89 | 160·6 | 104·1 | 0·7059 | − 9·33 | 0·9974 |
| LD | 107–6640 | U/l | 100 | 431·3 | 348·8 | 0·8494 | − 17·50 | 0·9964 |
| CK | 15–5140 | U/l | 77 | 263·2 | 283·0 | 1·0197 | 13·56 | 0·9961 |
| Cholesterol | 2·3–8·3 | mmol/l | 36 | 5·1 | 5·4 | 1·0844 | − 0·11 | 0·9949 |
| Triglyceride | 0·6–13·6 | mmol/l | 81 | 2·5 | 2·7 | 1·0384 | 0·19 | 0·9956 |
| Uric acid | 77·3–868·4 | μmol/l | 128 | 328·9 | 331·3 | 0·9555 | 17·25 | 0·9827 |

The bilirubin interference with bicarbonate and creatinine measurements has remained a major technical problem for which neither we nor the manufacturer has been able to provide a satisfactory solution. Samples with bilirubin levels greater than 171 µmol/l must be analysed on another instrument to obtain accurate results for bicarbonate and creatinine. In addition, bilirubin, hemolysis, and lipemia interfered at such significant levels with inorganic phosphorus and uric acid measurements that it was necessary to modify the original methods with sample blanking and changing of the secondary wavelength.

Electrolyte measurements on Demand have become a major area of concern for the authors' laboratory. Although the initial evaluation of the sodium, potassium, and chloride appeared acceptable, it has become apparent that the electrodes are not stable and require considerable trouble-shooting to correct drift. Of the three electrodes, the chloride electrode is the most unstable leading to anion gap calculation problems and repeat analysis of the sample. In addition, interferences due to unidentifiable substances result in values 10 to 15 mmol/l higher for chloride measurements indicated by inappropriate anion gap calculations and confirmed by repeat analysis on other instruments. Further, the routine weekly maintenance for the ISE module requires 1 to 1·5 h during which the instrument cannot be used for any analyses. A third problem is the slow turn-around time for urgent measurements caused by the need for recalibration of sodium, potassium, and chloride electrodes and the time that is required for the sample to reach the ISE module. Even by using the priority stat sampler cup (P cup) mechanism, the most rapid way to run any particular analysis, it takes 9·5 to 13·5 min from the beginning to the results being printed (this does not include the programming time). Finally, the bicarbonate test must be recalibrated every 4 h due to decreases in absorbance of the reagents once hydrated and placed in the instrument. This problem has led to falsely elevated levels of bicarbonate.

A problem encountered during this assessment was technical support for the Demand was less than the industry average. There was not a 24 hours a day, seven days a week 'hotline'. In addition, there was a lack of qualified service engineers and great difficulty in obtaining certain key replacement parts. Recently, Olympus Corporation (Lake Success, New York 11042, USA) has taken over full responsibility for service.

Our evaluation of the Demand found it to be precise, the linearities met the stated ranges except for BUN and total protein, and correlation of the 20 analytes was acceptable except for the triglyceride method which also measured free glycerol and was omitted from our test menu. It became the primary instrument of enzyme analysis because the increased linearity and good precision eliminated the large number of repeat analyses required by the SMAC I. The Demand is easy to operate; does not require a great deal of personnel training for the basic operation; and daily and weekly maintenance is minimal except for the ISE module. Calibration once per 24 h proved to be valid except for bicarbonate. The three major disadvantages of the Demand are the bilirubin interference with serum bicarbonate and creatinine measurements, slow turnaround time for urgent measurements of electrolytes, and the less than adequate technical support.

### Acknowledgements

We would like to acknowledge the expert technical assistance of the Automated Chemistry Section staff of the Clinical Laboratory of the UCLA Medical Center. We also thank Ms Jerry Turner for her expert assistance in preparation of this manuscript.

### References

1. Lauderdale, V., Haden, B., Baldwin, J., Eckfelt, J., Picka, C. and Jackson, B., *Clinical Chemistry*, **31** (1985), 917 (Abstract).
2. Kampa, I. S., Becker, J. and Zegiares, P., *Clinical Chemistry*, **31** (1985), 918 (Abstract).
3. The Demand System, *Operator's Manual* (Cooper Biomedical, Inc., Malvern, Pennsylvania 19355, USA).
4. Schotters, S. B., McBride, J. H., Rodgerson, D. O., McGinley, M. and Pisa, M. *Clinical Chemistry,* **32** (1986), 2109.
5. Davis, J. E., In *Clinical Chemistry Theory, Analysis, and Correlation* Eds. L. A. Kaplan and A. J. Pesce (C.V. Mosby Company, St. Louis, Toronto, Princeton, 1984), 264.
6. Glick, M. R., Ryder, K. W. and Jackson, S., *Clinical Chemistry*, **32** (1986), 470.

